# Development of a novel murine heart failure model overexpressing human renin and angiotensinogen

**DOI:** 10.1002/2211-5463.12810

**Published:** 2020-03-28

**Authors:** Tomoya Hara, Takeshi Yamamura, Mirei Murakami‐Asahina, Hirokazu Matsumoto, Michiyasu Takeyama, Ray Kanagawa, Tomoyuki Nishimoto

**Affiliations:** ^1^ Takeda Pharmaceutical Co Ltd Shonan Research Center Fujisawa Japan

**Keywords:** angiotensinogen, calsequestrin transgenic mouse, heart failure, imarikiren, renin, renin inhibitor

## Abstract

Renin is the rate‐limiting enzyme of the renin–angiotensin system cascade, which drives the pathophysiological progression of heart failure. Species differences in the amino acid sequence of the catalytic domain of renin limit evaluations of the potency and efficacy of human renin inhibitors in animal models, and a high dose of inhibitors is usually needed to show its organ‐protective effects in rodents. In the present study, we developed a novel murine heart failure model (triple‐tg) to enable us to evaluate the cardioprotective effect of renin inhibitors at more relevant doses for humans, by cross‐breeding calsequestrin transgenic (CSQ‐tg) mice with human renin and human angiotensinogen double‐transgenic mice. The triple‐tg mice exhibited increased plasma renin activity, worsened cardiac hypertrophy, and higher mortality compared to CSQ‐tg mice. Triple‐tg mice treated with 10 mg·kg^−1^ of TAK‐272 (imarikiren/SCO‐272), an orally active direct renin inhibitor, exhibited improvements in heart failure phenotypes, such as cardiac hypertrophy and survival rate; however, a dose of 300 mg·kg^−1^ was required to improve symptoms in CSQ‐tg mice. Our results suggest that this newly generated triple‐tg heart failure model is useful to evaluate the cardioprotective effects of human renin inhibitors at clinically relevant doses, thereby minimizing the concerns of off‐target effects related to much higher drug exposure than that achieved in clinical study.

## Graphical abstract

A high dose of human renin inhibitors is required to show efficacy in rodent models due to species differences between renin. We developed a novel murine heart failure model (triple‐tg) by cross‐breeding calsequestrin transgenic mice with human renin and human angiotensinogen double‐transgenic mice and investigated the cardioprotective effect of imarikiren at clinically relevant doses.

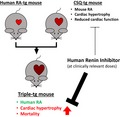

**Abbreviations**
ANP: atrial natriuretic peptide
BP: blood pressure
CSQ: calsequestrin
CSQ‐tg: calsequestrin transgenic
LV: left ventricle
NT‐proBNP: N‐terminal pro‐brain natriuretic peptide
PRA: plasma renin activity
RAS: renin–angiotensin system
RA‐tg: human renin and human angiotensinogen double‐transgenic
SBP: systolic blood pressure
Triple‐tg: calsequestrin, human renin, and human angiotensinogen triple‐transgenic
WT: wild‐type

The renin–angiotensin system (RAS) plays a key role in the pathophysiological progression of heart failure, which is one of the most common causes of death worldwide 1, 2. It is well known that RAS is activated in heart failure patients and involved in cardiac remodeling, apoptosis, and ventricular dilation 3, 4, 5, 6. Because the generation of angiotensin I from angiotensinogen, which is catalyzed by renin, is a rate‐limiting step in the RAS pathway, inhibition of renin should be an efficient way to reduce RAS activation 7, 8. TAK‐272 (imarikiren/SCO‐272) is a novel and orally effective renin inhibitor, which has shown potent and persistent inhibition of plasma renin activity (PRA) in healthy subjects in early clinical trials 9. The calsequestrin transgenic (CSQ‐tg) mouse is a heart failure model that has abnormal Ca^2+^ handling in cardiomyocytes due to the overexpression of CSQ, an important protein for cellular Ca^2+^ homeostasis. This yields various heart failure phenotypes including cardiac hypertrophy and low contractile function with premature death 10, 11, 12. We previously evaluated the cardioprotective effects of TAK‐272 in CSQ‐tg mice and found significant improvement in cardiac hypertrophy as well as mortality, although high doses were required for such significant effects. For TAK‐272 as well as other renin inhibitors optimized for human renin, generally high concentrations of the drugs are needed to exert inhibitory effects on rodent renin due to species differences in the amino acid sequence in the catalytic domain of renin 13, 14. In fact, the renin inhibitory activity of TAK‐272 in mice is 25 times weaker than that in humans 15. In addition, human renin cannot cleave rodent angiotensinogen efficiently, and vice versa 13, 16. Based on these findings, human renin and human angiotensinogen double‐transgenic (RA‐tg) animals have been used for *in vivo* evaluation of renin inhibitors against hypertension and kidney dysfunction to reduce the dose of renin inhibitors and exclude the possibility of off‐target effects related to high doses 17, 18. In this study, we generated human renin, human angiotensinogen, and canine calsequestrin transgenic (triple‐tg) mice, and then investigated the cardioprotective effect of TAK‐272 in this model in addition to its heart failure phenotypes.

## Materials and methods

### Animals

The transgenic DBA/2N mice with cardiac‐specific overexpression of canine CSQ were originally developed in a facility at the Indiana University School of Medicine 11 and then bred by Takeda Rabics (Osaka, Japan). First, transgenic mice carrying either human renin or human angiotensinogen were produced by injecting linear DNA fragments consisting of either the entire human renin (15.3 kbp) or entire human angiotensinogen (14 kbp) gene into C57BL/6J eggs. The double‐transgenic mice with systemic overexpression of human renin and human angiotensinogen (RA‐tg) were produced by breeding heterozygous human renin mice with heterozygous human angiotensinogen mice in Takeda Pharmaceutical Company (Kanagawa, Japan). CSQ, human renin, and human angiotensinogen triple‐transgenic (triple‐tg), CSQ‐tg, RA‐tg, and wild‐type (WT) mice used in this study were generated by breeding heterozygous CSQ‐tg mice with heterozygous RA‐tg mice. All these transgenic lines have a hybrid (DBA/2N × C57BL/6J) F1 background. Polymerase chain reaction (PCR) assay was conducted for genotyping by labchip gx software version 4.0.1418.0 (PerkinElmer, Waltham, MA, USA) using Tks Gflex™ DNA Polymerase (Takara Bio Inc., Kusatsu, Japan) with human renin primers (TTGGGAGCCAAGAAGAGGCTG and GCGCTGGTGAGCGTGTATTC, approx. 370 bp) and human angiotensinogen primers (AAAATTGAGCAATGACCGCATCAG and GCTTCAAGCTCAAAAAAAATGCTGTTC, approx. 930 bp) or canine CSQ primers (CTCTGACAGAGAAGCAGGCACTTTACATGG and GATGAACAGGTGTGTTCTCTTCAT, 407 bp) to confirm the expression of these genes; then, all the mice were distinguished as triple‐tg, CSQ‐tg, RA‐tg, or WT mice based on the genotypes. As both male and female mice showed comparable symptoms and survival rates in the preliminary studies, both sexes were used in this study due to limited animal availability. All animal experiments were conducted in accordance with EU Directive 2010/63/EU and approved by the Institutional Animal Care and Use Committee of Shonan Research Center, Takeda Pharmaceutical Company Limited.

### Characterization of cardiovascular parameters and PRA on transgenic mice

The systolic blood pressure (SBP) of male WT, RA‐tg, CSQ‐tg, and triple‐tg mice (*n* = 10 for each group) was measured by the tail‐cuff method using a BP‐98A blood pressure analyzer (Softron, Tokyo, Japan) under blind conditions at 4 weeks old. Blood samples were collected from the tail vein at 5 weeks of age in conscious state. EDTA was added as an anticoagulant, for a final concentration of 3 mm (15575020; Thermo Fisher Scientific K.K., Tokyo, Japan). The PRA was measured with a commercially available radioimmunoassay kit (Fujirebio, Tokyo, Japan) which measures total angiotensin I converted from human and mouse angiotensinogen by human and mouse renin, respectively. Hemodynamic parameters were also evaluated at 5 weeks old. Mice were anesthetized with 1–2% isoflurane (Mylan, Hatfield, UK). A catheter with a pressure sensor diameter of 1.4F, SPR‐671 (Millar Instruments, Houston, TX, USA), was inserted into the left ventricle through the right carotid artery. Left ventricular end‐diastolic pressure and the rates of intraventricular pressure rise (d*P/*d*t* max) and decline (d*P/*d*t* min) were measured under blind conditions. Heart rate as an indicator of depth of anesthesia was consistent among these animals (data not shown). These data were incorporated into PowerLab and analyzed with labchart v.8 and blood pressure module (ADInstruments, Sydney, New South Wales, Australia). After the measurement, blood samples were collected from the abdominal vein, and the hearts and lungs were excised and weighed. A heart failure biomarker, plasma N‐terminal pro‐brain natriuretic peptide (NT‐proBNP) levels were measured with a commercially available enzyme‐linked immunosorbent assay (ELISA) kit (SEA489Mu; Cloud‐Clone, Houston, TX, USA). In this NT‐proBNP assay, full‐length mouse NT‐proBNP peptide (custom‐made; Scrum, Tokyo, Japan) was used as the standard. The left ventricles (LV) were used for further histochemical and gene expression analyses. Upper half of LVs were fixed in Bouin solution at room temperature for 5 h and then embedded in paraffin for further staining. Total RNA was extracted from the leftover LVs using the RNeasy Mini Kit (Qiagen, Hilden, Germany) and converted into cDNA using the High‐Capacity cDNA Reverse Transcription Kit (Life Technologies, Carlsbad, CA, USA). The gene expression was analyzed on a 7900HT Fast Real‐Time PCR System (Life Technologies) using the TaqMan^®^ Universal Master Mix II (Life Technologies) and primer–probe sets (TaqMan^®^ Gene Expression Assays, Life Technologies) against atrial natriuretic peptide (*ANP*, Mm01255747_g1) and β‐actin (ACTB, Mm02619580_g1). ACTB was used as an endogenous control gene. The relative gene expression values were calculated by the ΔΔCT method.

### Difference in survival rate between CSQ‐tg and triple‐tg mice

The survival rate of male CSQ‐tg (*n* = 43) and triple‐tg mice (*n* = 41) was monitored for 68 days. As a humane endpoint, the animals were euthanized in cases where rectal temperature dropped (below 29 °C), body weight decreased severely, or extremely low activity was observed according to prespecified criteria 15. Body weight was measured twice a week, and rectal temperature was also measured once daily until the end of the study.

### Drugs

TAK‐272 was synthesized by Takeda Pharmaceutical Company (Kanagawa, Japan), and aliskiren was purchased from KNC Laboratories (Kobe, Japan). These compounds were dissolved in 0.5% (w/v) methylcellulose solution, and drug solutions were administered by oral gavage once daily.

### Responsiveness of triple‐tg mice to human renin inhibitors

The female triple‐tg mice at 4 weeks old were treated with vehicle (*n* = 10) or TAK‐272 (10 mg·kg^−1^, *n* = 9) once daily for 2 weeks to evaluate the effects of TAK‐272 on blood pressure (BP). The SBP was measured 2 and 24 h after dosing on day 7. Blood was collected 5 days after BP measurement for plasma NT‐proBNP measurement. One mouse from the TAK‐272‐treated group died before BP measurement. Two mice in the vehicle‐treated group died before blood collection. The hearts were excised and used for RNA extraction. In another experiment, the female triple‐tg mice were treated with vehicle (*n* = 6) or TAK‐272 (10 mg·kg^−1^, *n* = 6). Blood samples were collected pretreatment, 2, and 24 h after a single administration, and their PRA was measured. All procedures were conducted as described in Characterization of cardiovascular parameters and PRA on transgenic mice.

### Cardioprotective effect of TAK‐272 in triple‐tg mice

Six WT mice (male: 3; female: 3) and nine triple‐tg mice (male: 5; female: 4) per treatment group were used to investigate the cardioprotective effects of TAK‐272. WT mice were treated with vehicle, and triple‐tg mice were treated with vehicle or TAK‐272 at 3 or 10 mg·kg^−1^ once daily from 4 weeks of age. Blood samples were collected pretreatment, 2, 6, and 24 h after the first dose. PRAs were measured as described in Characterization of cardiovascular parameters and PRA on transgenic mice. SBP was evaluated at 2 and 24 h on day 6 after oral administration following the method as described in Characterization of cardiovascular parameters and PRA on transgenic mice. After 2 weeks of treatment, the animals were euthanized; then, the hearts and lungs were excised and weighed. A total of six triple‐tg mice (three in vehicle, two in TAK‐272 at 3 mg·kg^−1^, and one in TAK‐272 at 10 mg·kg^−1^) died before the study end. In a separate animal setting, the effect of 10 mg·kg^−1^ of TAK‐272 on survival rate was evaluated in triple‐tg mice. Twenty triple‐tg mice (male: 8; female: 12) were treated with vehicle or TAK‐272 once daily starting at 4 weeks of age for 24 days. Survival rate was monitored as described in Difference in survival rate between CSQ‐tg and triple‐tg mice.

### Cardioprotective dose of TAK‐272 in CSQ‐tg mice

To find the cardioprotective dose of TAK‐272 in CSQ‐tg mice, five female WT mice or 10 female CSQ‐tg mice were used for each treatment group. WT mice were treated with vehicle, and CSQ‐tg mice were treated with vehicle or TAK‐272 at 100 or 300 mg·kg^−1^ from 4 weeks of age. After 2 weeks of treatment, the animals were euthanized; then, the hearts and lungs were excised and weighed.

### Statistical analysis

All statistical analyses were performed using exsus software (version 8.0.0; CAC Croit, Tokyo, Japan). For comparison between two groups, homogeneity of variance was analyzed by *F*‐test first; then, Student's *t*‐test (*P *> 0.20 by *F*‐test) or Aspin–Welch *t*‐test (*P* < 0.20 by *F*‐test) was performed. For comparison between more than three groups, homogeneity of variance was analyzed by Bartlett's test at first; then, one‐way ANOVA followed by Dunnett's test or Williams' test (*P *> 0.05 by Bartlett's test), or Kruskal–Wallis nonparametric analysis of variance followed by Steel's test or Shirley–Williams test (*P* < 0.05 by Bartlett's test) was performed. All data are expressed as the mean ± SD. The Kaplan–Meier method with log‐rank test was used for survival analysis.

## Results

### Increased PRA and BP in triple‐tg mice

**Figure 1:**
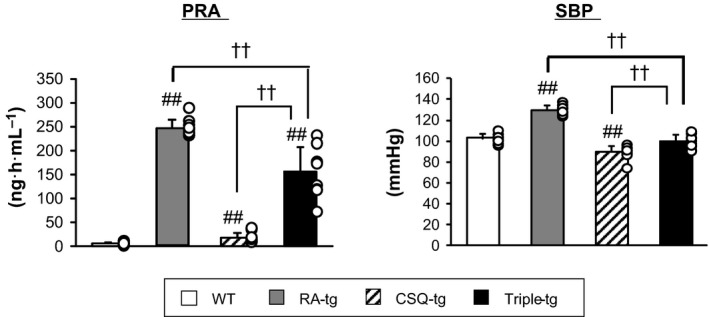
The baseline characteristics of PRA and BP of triple‐tg mice. The baseline characteristics of PRA and SBP of WT, RA‐tg, CSQ‐tg, and triple‐tg mice (*n* = 9–10 per group). ##*P* < 0.01 vs. WT, and ^††^ *P* < 0.01 vs. triple‐tg by Dunnett's test or Steel's test. The error bars represent SD.

Overexpression of human renin and human angiotensinogen in WT mice resulted in a remarkable increase in PRA and SBP (Fig. 1). Both PRA and SBP were consistently elevated in triple‐tg mice compared to levels in CSQ‐tg mice, whereas CSQ‐tg mice had slightly higher PRA than WT mice.

### Heart failure parameters in triple‐tg mice

**Figure 2:**
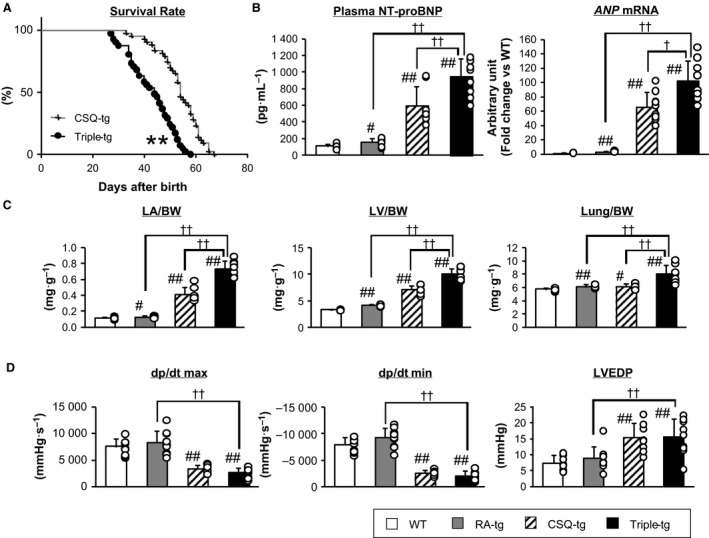
Heart failure parameters of triple‐tg mice. (A) Survival curves of CSQ‐tg (*n* = 43) and triple‐tg mice (*n* = 41). **P < 0.01 vs. CSQ‐tg mice by Kaplan–Meier survival analysis with a log‐rank test. (B) Plasma NT‐proBNP levels and cardiac *ANP* mRNA expression levels, (C) heart and lung weights standardized by body weight (BW), and (D) hemodynamic parameters of WT, RA‐tg, CSQ‐tg, and triple‐tg mice (*n* = 8–10 per group). ^#^ *P* < 0.05, ^##^ *P* < 0.01 vs. WT, and ^†^ *P* < 0.05, ^††^ *P* < 0.01 vs. triple‐tg by Dunnett's test or Steel's test. The error bars represent SD.

Survival rate was compared between CSQ‐tg mice and triple‐tg mice. The median survival time of triple‐tg mice was shorter than that of CSQ‐tg mice (Fig. 2A, CSQ‐tg: 54 and triple‐tg: 44 days). We did not monitor the survival rate of RA‐tg mice in the same study; however, we have confirmed that RA‐tg mice survive for 10 weeks at least without any health concerns. It is reported that 6‐ to 8‐month‐old human renin and human angiotensinogen transgenic mice were eligible for blood pressure and PRA measurement 13, 14. Plasma NT‐proBNP level, cardiac *ANP* mRNA expression level, and cardiac hypertrophy of the four strains were evaluated (Fig. 2B,C). Cardiac hypertrophy and lung weight were increased in CSQ‐tg mice compared to those in WT mice. Although RA‐tg mice showed only slight increases in left atrium, LV, and lung weight, triple‐tg mice showed severely increased cardiac and lung weight accompanied by elevated plasma NT‐proBNP level. Cardiac *ANP* mRNA expression level of triple‐tg mice was also slightly increased than that of CSQ‐tg mice. LV sections of the four strains also demonstrated severe cardiac hypertrophy in triple‐tg mice (Fig. S1). However, cardiac hemodynamic parameters were not deteriorated in triple‐tg mice compared to those in CSQ‐tg mice (Fig. 2D). Similarly, there was no clear difference between CSQ‐tg and triple‐tg mice on cardiac fibrosis severity, although slightly increased cardiac fibrosis was observed in CSQ‐tg and triple‐tg mice compared to WT or RA‐tg mice (Fig. S1).

### Cardioprotective effects of TAK‐272 in CSQ‐tg or triple‐tg mice

**Figure 3:**
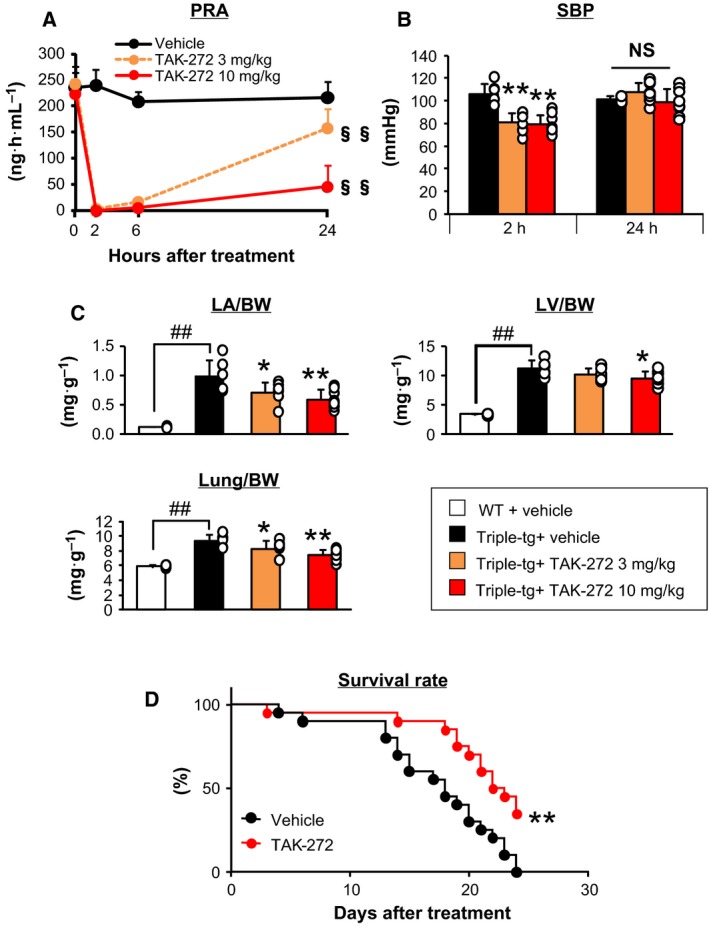
Cardioprotective effects of TAK‐272 in triple‐tg mice. (A) PRA inhibition over 24 h after a single oral administration of TAK‐272 at 3 or 10 mg·kg^−1^ in triple‐tg mice (*n* = 9 per group). (B) The SBP of triple‐tg mice measured at 2 and 24 h on day 6 after oral administration of vehicle or TAK‐272 at 3 or 10 mg·kg^−1^ once daily (*n* = 6–8 per group). **P < 0.01 vs. vehicle Williams' or Shirley–Williams test. (C) Heart and lung weights, standardized by BW, of WT or triple‐tg mice orally treated with either vehicle or TAK‐272 (3 or 10 mg·kg^−1^) for 2 weeks (*n* = 6–8 per group). ^§§^ *P* < 0.01 vs. pretreatment by paired *t*‐test with Bonferroni's correction, ^##^ *P* < 0.01 vs. WT by Student's *t*‐test or Aspin–Welch test, *P < 0.05 and **P < 0.01 vs. triple‐tg + vehicle by Williams' test or Shirley–Williams test. (D) Survival curves of triple‐tg mice orally treated with either vehicle or TAK‐272 at 10 mg·kg^−1^ (*n* = 20 per group) **P < 0.01 vs. vehicle by Kaplan–Meier survival analysis with a log‐rank test. The error bars represent SD.

TAK‐272 at both 3 and 10 mg·kg^−1^ completely inhibited PRA for 6 h, and 10 mg·kg^−1^ showed more persistent PRA inhibition over 24 h after a single administration in triple‐tg mice (Fig. 3A). TAK‐272‐treated groups showed a transient decrease in SBP at 2 h after treatment, and the decreased BP was restored at 24 h (Fig. 3B). Both cardiac hypertrophy and lung weight were decreased in a dose‐dependent manner with TAK‐272 at 3 or 10 mg·kg^−1^ in triple‐tg mice (Fig. 3C), while a much higher dose (300 mg·kg^−1^) of TAK‐272 was required to decrease LV weight in CSQ‐tg mice. The ratios of LV weight to body weight (mg·g^−1^) in CSQ‐tg mice were 7.1 ± 0.6 for vehicle, 6.7 ± 0.5 for TAK‐272 at 100 mg·kg^−1^, and 6.4 ± 0.3 for TAK‐272 at 300 mg·kg^−1^ (*P* < 0.05, decrease in LA or lung weight was not statistically significant). Although hemodynamic parameters of triple‐tg mice were not augmented by TAK‐272 at 10 mg·kg^−1^ (Fig. S2), this treatment significantly improved their mortality (Fig. 3D), consistent with the improvement of cardiac hypertrophy. The median survival times of the vehicle‐treated and TAK‐272‐treated groups were 18 and 22.5 days, respectively. Reduction of plasma NT‐proBNP level and cardiac *ANP* mRNA expression level by TAK‐272 treatment at 10 mg·kg^−1^ was not statistically significant (Fig. S3), whereas *NOX4* mRNA expression level was decreased by the TAK‐272 treatment (Fig. S4).

## Discussion

It is ideal to investigate the efficacy of drugs using clinically relevant doses (or less) in preclinical study to minimize the concerns of off‐target effects related to much higher doses, which can affect the results. However, the amino acid sequence differences in the catalytic domain of renin between humans and rodents generally require higher doses of human renin inhibitors to show effects in rodent disease models 13, 14. To overcome this concern, we generated a heart failure animal model overexpressing human renin and human angiotensinogen by breeding CSQ‐tg mice with RA‐tg mice. CSQ‐tg mice have lower SBP than WT mice. We thought that is the reason why triple‐tg mice had lower SBP compared to RA‐tg mice. However, triple‐tg mice showed higher SBP compared to CSQ‐tg mice as we expected as well as PRA. This newly developed triple‐tg heart failure mouse showed higher mortality accompanied by severe cardiac hypertrophy than CSQ‐tg mice. In CSQ‐tg mice, 300 mg·kg^−1^ of TAK‐272 was required to exert an antihypertrophic effect shown as decrease in LV weight in the present study. Our previous study also indicated that TAK‐272 at 300 mg·kg^−1^ improved the survival rate of CSQ‐tg mice, but this effect was not significant at 100 mg·kg^−1^ 15. On the other hand, we demonstrated that a much lower dose of TAK‐272 (10 mg·kg^−1^) significantly improved cardiac hypertrophy and mortality in triple‐tg mice in the present study. In early human clinical trials, doses of 5–200 mg of TAK‐272 were tested in healthy subjects. TAK‐272 at 5 mg per person demonstrated potent and prolonged renin inhibition over 24 h, which was comparable to the effects of 10 mg·kg^−1^ in triple‐tg mice and the effects of 300 mg·kg^−1^ in CSQ‐tg mice, as shown in our previous study 9, 15. This indicates that the effective dose of TAK‐272 in triple‐tg mice was much closer to a clinical dose in humans, than in CSQ‐tg mice. This finding suggests that the triple‐tg mouse is useful to minimize the concerns of off‐target effects by reducing the effective dose. It also suggests that the cardioprotective effect in triple‐tg mice was induced purely by on‐target effects.

As a result of the increased susceptibility for human renin inhibition, transient but obviously lowered SBP was observed 2 h after a single administration of TAK‐272. We cannot exclude that the afterload reduction induced by decreasing BP was possibly involved in the mechanism of the cardioprotective effect of TAK‐272. In addition, 2 weeks of treatment with TAK‐272 at 10 mg·kg^−1^ decreased *NOX4* mRNA expression level in triple‐tg mice consistently with our previous study in CSQ‐tg mice 15. Reduced levels of reactive oxygen species could also contribute to its cardioprotective effect. On the other hand, a decrease in plasma NT‐proBNP level and cardiac ANP mRNA expression level by TAK‐272 treatment at 10 mg·kg^−1^ was not significant in this study. Not only triple‐tg mice, but also CSQ‐tg mice presented much higher levels compared to WT mice. Based on these findings, it is possible that their increased levels in triple‐tg mice are not so much dependent on renin activity and that could be the reason why significant restoration in these markers was not observed with TAK‐272 treatment. Moreover, echocardiographic analysis may reveal more insights of structural characteristics of this animal model and the effects of TAK‐272 treatment in the future. Further experiments will be needed to fully elucidate the mechanism of its cardioprotective effect in triple‐tg mice.

One limitation of this study is the PRA level of triple‐tg mice. Although the PRA of WT or CSQ‐tg mice was comparable or slightly higher than that in healthy subjects and heart failure patients 19, 20, triple‐tg mice had much higher PRA due to overexpression of human renin and human angiotensinogen. Therefore, caution is required in interpreting the clinical translatability of the relationship between PRA inhibition and the cardioprotective effects induced by TAK‐272 in triple‐tg mice.

In conclusion, we successfully generated a heart failure mouse model which has cardiac hypertrophy and higher mortality with increased susceptibility to human renin inhibition. Our findings indicate that this newly developed triple‐tg mouse enables us to use clinically relevant doses to investigate cardioprotective effects of human renin inhibitors with minimized concerns of the off‐target effects related to higher doses. This study also suggests that generating rodent models that have the human protein should be considered in cases the drug target protein has a species difference, similar to renin in this example. This could be a useful strategy to evaluate the efficacy of drugs to accelerate drug discovery.

## Conflict of interest

All authors were employees of Takeda Pharmaceutical Company Limited when this study was conducted. This manuscript describes a research molecule, TAK‐272 (imarikiren/SCO‐272), that is being developed by SCOHIA PHARMA, Inc.

## Author contributions

TH, RK, and TN conceived the study; HM, MT, RK, and TN supervised the study; TH and TN designed experiments; TH, TY, and MM‐A performed experiments; TH and TY analyzed the data; TH wrote the manuscript; and MT and TN made manuscript revisions.

## Supporting information


**Fig. S1.** Cardiac fibrosis in triple‐tg mice. Representative Sirius red‐stained left ventricular sections of WT, RA‐tg, CSQ‐tg, and triple‐tg mouse. The study detail is described in section 2.2. Paraffin‐embedded left ventricle tissues were sliced into 3 µm sections at papillary muscle level and placed onto saline‐coated glass slides. These sections were stained with picrosirius red, then their images were captured with NanoZoomer S360 (Hamamatsu Photonics K.K., Japan). The length of the scale bar in low magnification images: 2.5 mm, high magnification images: 100 µm.

**Fig. S2.** Hemodynamic parameters of triple‐tg mice after TAK‐272 treatment. Hemodynamic parameters of triple‐tg mice orally treated either vehicle (n = 5) or TAK‐272 at 10 mg·kg^−1^ for two weeks (n = 8). The study detail is described in section 2.5. There were originally 10 mice for vehicle group and 9 mice for TAK‐272 group at start point. Two mice in vehicle group and one mouse in TAK‐272 group died before the hemodynamic measurement, and 3 mice in vehicle group were unavailable for the evaluation due to their health condition. The error bars represent SD.

**Fig. S3.** The effects of TAK‐272 on plasma NT‐proBNP levels and cardiac *ANP* mRNA expression levels in triple‐tg mice. Plasma NT‐proBNP levels and cardiac *ANP* mRNA expression levels in triple‐tg mice treated with vehicle or TAK‐272 at 10 mg·kg^−1^ for two weeks (n = 8 per group). The study detail is described in section 2.5. The error bars represent SD.

**Fig. S4.** Cardiac *NOX4* mRNA expression levels in triple‐tg mice. (A) Cardiac *NOX4* mRNA expression levels of WT, RA‐tg, CSQ‐tg, and triple‐tg mice (n = 8‐10 per group). The study detail is described in section 2.2. ^#^ < 0.05, ^##^ *P* < 0.01 vs. WT by Dunnett's test or Steel's test, ^††^ *P* < 0.01 vs. triple‐tg by Dunnett's test or Steel's test. (B) Cardiac *NOX4* mRNA expression levels of triple‐tg mice orally treated with vehicle or TAK‐272 at 10 mg·kg^−1^ for two weeks (n = 8 per group). The study detail is described in section 2.5. *P < 0.05 vs. vehicle by Student's t‐test. Primer‐probe sets against NADPH oxidase 4 (*NOX4*, Mm00479246_m1) was used for gene expression analyses, and ACTB was used as an endogenous control gene. The error bars represent SD.
