# Supplementary figures and images for: Development of a novel murine heart failure model overexpressing human renin and angiotensinogen
Source: FEBS Open Bio. 2020 Mar 28;10(5):718–25. doi: 10.1002/2211-5463.12810 (PMC7193172; doi:10.1002/2211-5463.12810)

**Non-tg**

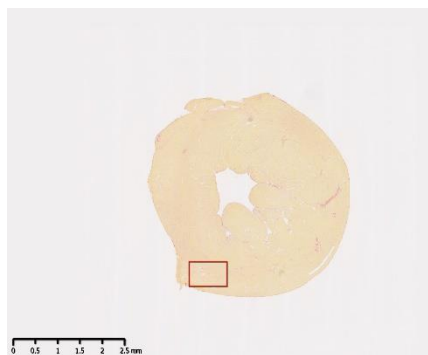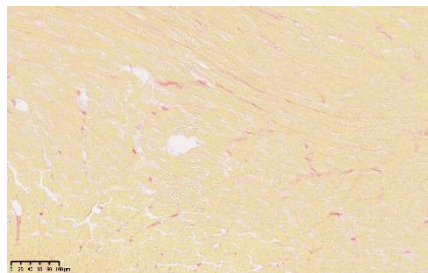

**RA-tg**

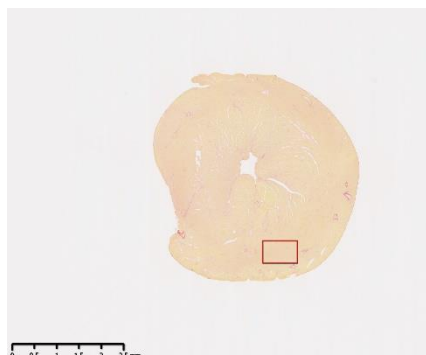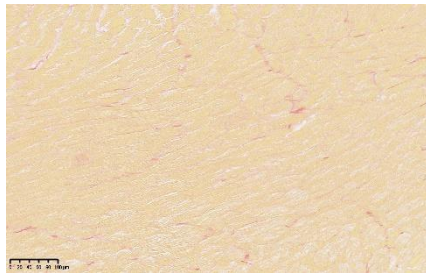

**CSQ-tg**

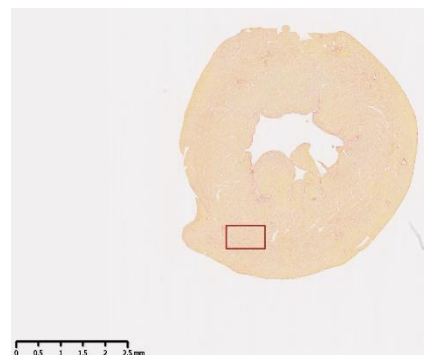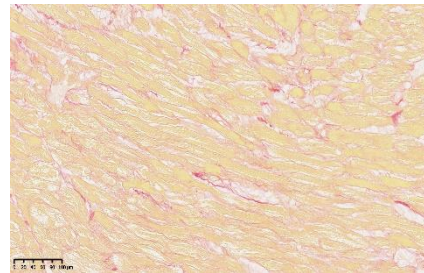

**Triple-tg**

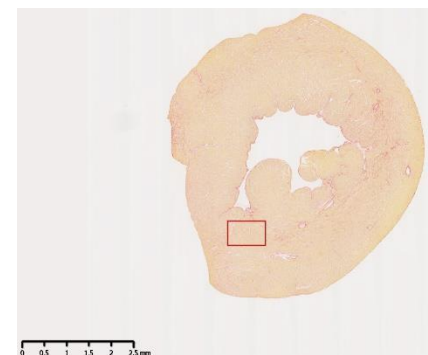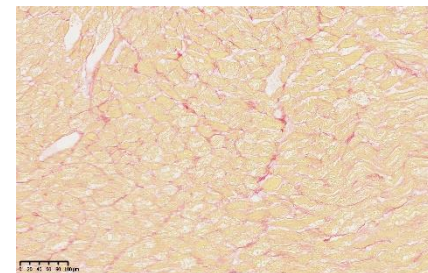

Supplement: Supplementary file 1 — Fig. S1. Cardiac fibrosis in triple‐tg mice. Representative Sirius red‐stained left ventricular sections of WT, RA‐tg, CSQ‐tg, and triple‐tg mouse. The study detail is described in section 2.2. Paraffin‐embedded left ventricle tissues were sliced into 3 µm sections at papillary muscle level and placed onto saline‐coated glass slides. These sections were stained with picrosirius red, then their images were captured with NanoZoomer S360 (Hamamatsu Photonics K.K., Japan). The length of the scale bar in low magnification images: 2.5 mm, high magnification images: 100 µm. [file FEB4-10-718-s001.pdf]

***dP/dt max***

**NS**

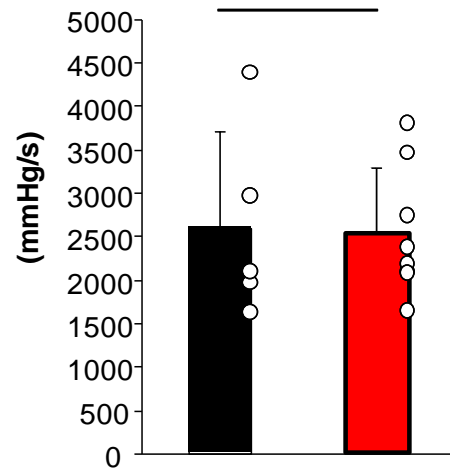

***dP/dt min***

**NS**

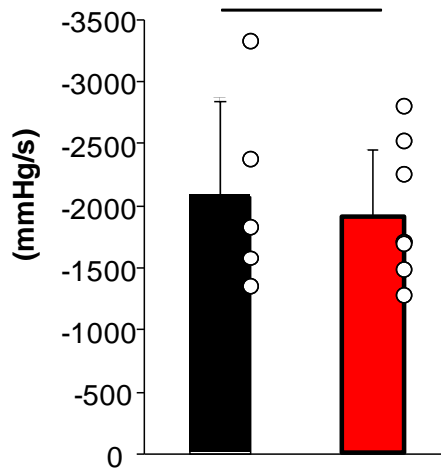

**LVEDP**

**NS**

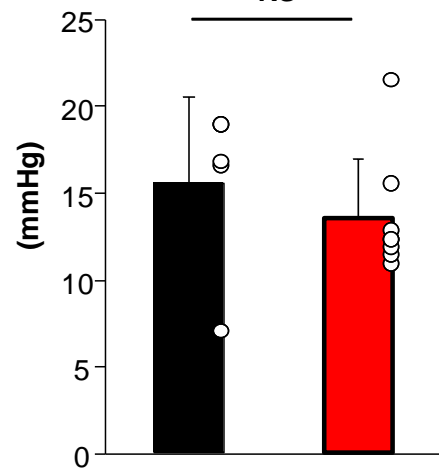

■ Vehicle

■ TAK-272 10 mg/kg

Supplement: Supplementary file 2 — Fig. S2. Hemodynamic parameters of triple‐tg mice after TAK‐272 treatment. Hemodynamic parameters of triple‐tg mice orally treated either vehicle (n = 5) or TAK‐272 at 10 mg·kg−1 for two weeks (n = 8). The study detail is described in section 2.5. There were originally 10 mice for vehicle group and 9 mice for TAK‐272 group at start point. Two mice in vehicle group and one mouse in TAK‐272 group died before the hemodynamic measurement, and 3 mice in vehicle group were unavailable for the evaluation due to their health condition. The error bars represent SD. [file FEB4-10-718-s002.pdf]

## Plasma NT-proBNP

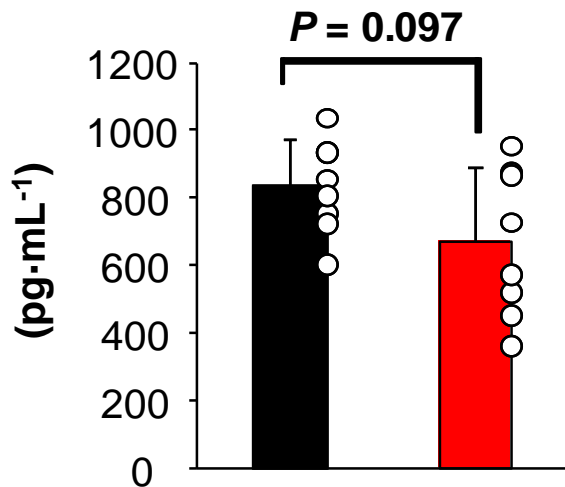

## Cardiac ANP mRNA

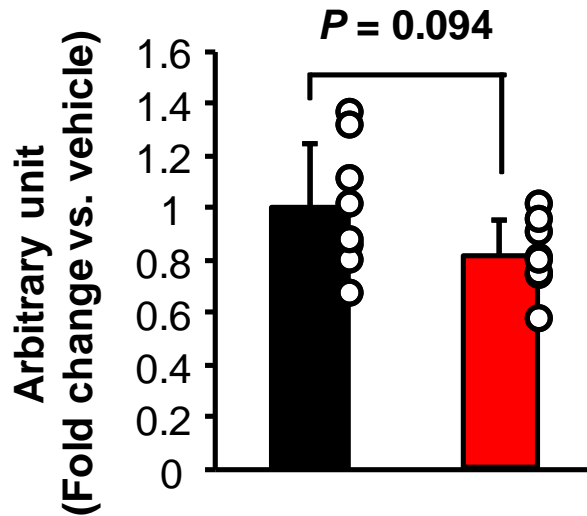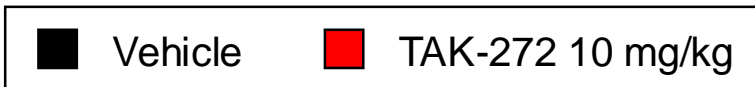

Supplement: Supplementary file 3 — Fig. S3. The effects of TAK‐272 on plasma NT‐proBNP levels and cardiac ANP mRNA expression levels in triple‐tg mice. Plasma NT‐proBNP levels and cardiac ANP mRNA expression levels in triple‐tg mice treated with vehicle or TAK‐272 at 10 mg·kg−1 for two weeks (n = 8 per group). The study detail is described in section 2.5. The error bars represent SD. [file FEB4-10-718-s003.pdf]

(A)

**NOX4 mRNA**

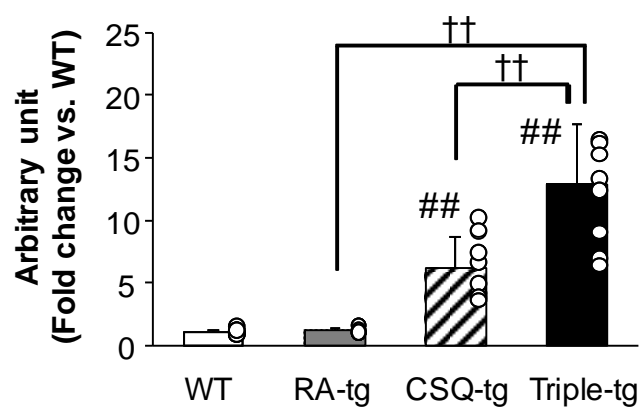

(B)

**NOX4 mRNA (triple-tg)**

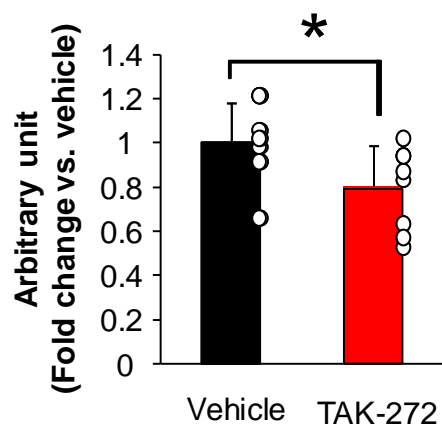

Supplement: Supplementary file 4 — Fig. S4. Cardiac NOX4 mRNA expression levels in triple‐tg mice. (A) Cardiac NOX4 mRNA expression levels of WT, RA‐tg, CSQ‐tg, and triple‐tg mice (n = 8‐10 per group). The study detail is described in section 2.2. # < 0.05, ## P < 0.01 vs. WT by Dunnett's test or Steel's test, †† P < 0.01 vs. triple‐tg by Dunnett's test or Steel's test. (B) Cardiac NOX4 mRNA expression levels of triple‐tg mice orally treated with vehicle or TAK‐272 at 10 mg·kg−1 for two weeks (n = 8 per group). The study detail is described in section 2.5. *P < 0.05 vs. vehicle by Student's t‐test. Primer‐probe sets against NADPH oxidase 4 (NOX4, Mm00479246_m1) was used for gene expression analyses, and ACTB was used as an endogenous control gene. The error bars represent SD. [file FEB4-10-718-s004.pdf]
